# Immune-Related Adverse Events and Corticosteroid Use for Cancer-Related Symptoms Are Associated With Efficacy in Patients With Non-small Cell Lung Cancer Receiving Anti-PD-(L)1 Blockade Agents

**DOI:** 10.3389/fonc.2020.01677

**Published:** 2020-09-07

**Authors:** Mariona Riudavets, Joaquin Mosquera, Rosario Garcia-Campelo, Jorgina Serra, Georgia Anguera, Pablo Gallardo, Ivana Sullivan, Andrés Barba, Luís del Carpio, Agustí Barnadas, Ignasi Gich, Margarita Majem

**Affiliations:** ^1^Department of Medical Oncology, Hospital de la Santa Creu i Sant Pau, Barcelona, Spain; ^2^Department of Medicine, Universitat Autónoma de Barcelona (UAB), Barcelona, Spain; ^3^Department of Medical Oncology, Hospital Universitario a Coruña, a Coruña, Spain; ^4^Department of Clinical Epidemiology and Public Health, Hospital de la Santa Creu i Sant Pau, Barcelona, Spain; ^5^CIBER Epidemiología y Salud Pública (CIBERESP), Madrid, Spain; ^6^Sant Pau Biomedical Research Institute (IIB Sant Pau), Barcelona, Spain

**Keywords:** immune-related adverse events, immunotherapy, advanced NSCLC, corticosteroids, efficacy

## Abstract

**Background:** Immune-related adverse events (irAEs) have been associated with improved efficacy in advanced non-small cell lung cancer (NSCLC) patients receiving anti-PD-(L)1 blockade agents, while the concurrent use of corticosteroids seems to worsen it. We evaluated outcomes in advanced NSCLC patients treated with anti-PD-(L)1 blockade agents in relation to the presence of irAEs and the reasons for using corticosteroids: whether for palliative cancer-related reasons or for the management of irAEs.

**Methods:** Clinical outcomes in advanced NSCLC patients treated with anti-PD-(L)1 blockade agents were calculated with regard to the presence of irAEs and the use of corticosteroids. A landmark analysis was performed to avoid immortal time bias due to the time-dependent nature of irAEs.

**Results:** Out of a total of 267 patients, the 56.9% of patients who experienced irAEs had significantly improved outcomes. In the landmark analysis, median progression-free survival (PFS) was 12.4 months for patients with irAEs vs. 4.1 months for patients without irAEs (*p* < 0.001), while median overall survival (OS) was 28.2 vs. 12.5 months, respectively (*p* < 0.001). Likewise, objective response and disease control rates were significantly higher in patients experiencing irAEs: 48.6 vs. 22.8% and 77.1 vs. 39.6% (*p* < 0.001), respectively. Median OS was significantly shorter for patients receiving ≥10 mg of prednisone equivalent daily for cancer-related symptoms than for the rest of patients (<10 mg prednisone equivalent daily or for management of irAEs): 6 vs. 15.9 months (*p* < 0.001).

**Conclusions:** IrAEs were associated with improved efficacy in advanced NSCLC patients when a landmark analysis was applied. Patients receiving corticosteroids had significantly poorer outcomes when they were used for cancer-related symptoms.

## Introduction

Immunotherapy has become established as a new standard-of-care for multiple solid malignancies, including non-small cell lung cancer (NSCLC). Among other strategies, immune-checkpoint blockade agents targeting the inhibitory pathways of the immune cascade have resulted in an increase in the response against tumor cells (1, 2).

Examples of these agents are nivolumab and pembrolizumab, monoclonal antibodies against the programmed cell death-1 receptor (PD-1) and atezolizumab, durvalumab, and avelumab, against its ligand PD-L1. Most of these agents have been approved in different settings for the treatment of patients with NSCLC (3–8), which has led the scientific community to advance by exploring combinations of anti-PD-(L)1 blockade agents with chemotherapy and/or other immune-checkpoint blockade to achieve better results (9–11).

Due to their mechanism of action, anti-PD-(L)1 blockade agents can induce inflammatory side effects known as immune-related adverse events (irAEs), which are not triggered by conventional cytotoxic anticancer agents. The most commonly reported irAEs are those affecting the skin, the gastrointestinal tract and the thyroid gland, though any organ or system may be involved, including the lung, liver, and the hypophysis (12) IrAEs are generally mild, but ~10% of cases are severe and may require immunosuppressors and/or treatment discontinuation (13, 14).

However, immunotherapy raises several questions that remain unknown. First, a positive correlation between the presence of irAEs and the efficacy of immunotherapy has been postulated, suggesting that a proper management of such events might be required to maximize the therapeutic effect of these drugs and to avoid treatment interruption (15–23). Second, the activity of anti-PD-(L)1 blockade agents in patients receiving corticosteroids or antibiotics during immunotherapy is controversial, and their use and safety in certain groups, such as patients with brain metastasis, needs to be defined (24–26).

We performed a retrospective study to investigate irAEs profiles and their association with clinical activity in patients with advanced NSCLC treated with anti-PD-(L)1 blockade agents using landmark and multivariable analyses. Additionally, we evaluated the efficacy of immunotherapy with regard to the use of corticosteroids and antibiotics.

## Materials and Methods

The medical records of all patients with advanced NSCLC treated with anti-PD-(L)1 blockade agents at two tertiary institutions in Spain between March 2013 and August 2018 were reviewed. All patients starting an anti-PD-(L)1 blockade agent alone or in combination with chemotherapy or an anti-CTLA-4 (cytotoxic T-lymphocyte antigen 4) in any treatment line were included.

The end of follow-up was December 31, 2018. The study was approved by the local institutional review board.

Patients were evaluated for objective response rate (ORR), disease control rate (DCR), duration of response (DoR), progression-free survival (PFS), and overall survival (OS). Tumor responses were assessed as per clinical practice using the Response Evaluation Criteria for Solid Tumors (RECIST) version 1.1 every 8–12 weeks (27).

IrAEs were defined as adverse events with a potential immunologic basis that required close monitoring and/or potential intervention with immunosuppressives or hormone replacement. Patient symptoms and physical exploration and laboratory data were assessed at every cycle. Thyroid function was evaluated at baseline and every 6 weeks thereafter. irAEs severity was graded according to the Common Terminology Criteria for Adverse Events (CTCAE) version 4.0, as part of routine clinical practice (28).

Patient data were obtained from a unified database in which clinical and pathological characteristics and toxicity were accurately recorded.

ORR data included patients with partial or complete response, and DCR data included partial response, complete response, and stable disease. PFS and OS were measured as the time from the start of anti-PD-(L)1 blockade agent to documented disease progression or death owing to any cause (PFS) or to death (OS). Patients with no events were censored on the date of the last follow-up. Those patients who were not evaluable for response were not included in the ORR assessment but were included in the PFS and OS evaluations.

Corticosteroid usage within 1 month before the initiation or during anti-PD-(L)1 therapy, and the administration of antibiotics from 3 months before the start of anti-PD-(L)1 therapy to 3 months after finishing were also recorded. The reason for corticosteroid use was specified, distinguishing between management of irAEs or the palliative treatment of cancer-related symptoms. Types of corticosteroid and the prednisone equivalent daily dose, as well as type of antibiotics, were also collected. Transient corticosteroids given along with a chemotherapy combination were not registered.

To analyze efficacy according to the dose of corticosteroids patient were divided into two groups: those receiving prednisone equivalent ≥10 mg daily and those receiving prednisone equivalent ≤10 mg daily (including patients that did not receive corticosteroids).

### Statistical Analysis

Survival curves were estimated using the Kaplan-Meier method and compared with the log-rank test. Taking into account the immortal time bias due to the time-dependent nature of irAEs, we performed tests at 2.4-months for PFS and 5.9-months for OS landmark analyses including only patients manifesting disease control or those who were alive at these time points. Consequently, 92 patients were excluded from the PFS landmark analysis (*n* = 175) and 100 patients were excluded from the OS analysis (*n* = 167). Landmark times were pre-defined before the start of data analysis and were determined from the median PFS and OS data of patients with no irAEs (29, 30). In addition, 6 and 12-month landmark analyses were also performed as complementary evaluations.

Odds ratios were used for ORR and DCR. Univariable and multivariable Cox proportional hazard regression models were adopted to determine hazard ratios (HR).

Two multivariable analyses were performed. First, to determine the influence of clinical characteristics (such as age, smoking status, Eastern Cooperative Oncology Group [ECOG] performance status [PS], brain, and liver metastases, presence of irAEs, toxicity grade, use of prednisone equivalent ≥10 mg daily, use of antibiotics and treatment line), and second, to assess the influence of different types of irAEs on OS.

To describe our population, numbers and percentages were used for qualitative variables, while medians and interquartile ranges (IQR) were calculated for ordinal and quantitative variables with an asymmetric distribution.

All *p-*values were based on a two-sided hypothesis, and those <0.05 were considered statistically significant. Statistical analyses were carried out using IBM SPSS Statistics for Windows, version 25.0 (IBM SPSS Inc., Chicago, IL, USA).

## Results

### Patient Characteristics

We included 267 consecutive patients with advanced NSCLC treated with anti-PD-(L)1 blockade agents. Baseline characteristics of patients are described in Table 1. The median age was 66.1 years (range 26.7–85.2, IQR 14.3), 69.7% were male and 30.3% female. The majority of patients were current or former smokers (74.2%) and baseline ECOG PS was 0–1 in 85% of patients. Non-squamous was the most common histology (67.8%). Brain metastases were present in 15.7% of patients and liver metastases in 15.4%. PD-L1 expression analysis in tumor samples was available from 135 patients (50.6%), and expression was low (PD-L1 expression 1–49%) in 52 (38.5%), high (PD-L1 ≥ 50%) in 41 (30.4%), and negative (PD-L1 < 1%) in 42 (31.1%).

**Table 1:** Patients characteristics and comparison and the presence of irAEs.

**Category**	**Total *n* = 267 (%)**	**irAEs *n* = 152 (%)**	**No irAEs *n* = 115 (%)**	***P*-value**
**Gender** Male Female	186 (69.7) 81 (30.3)	108 (71.1) 44 (28.9)	78 (67.8) 37 (32.2)	0.593
**Age** median (range)	66.1 years (26.7–85.2, IQR 14.3)	66.4 years (26.7–85.2, IQR 15.3)	65.8 years (38.8–81.0, IQR 13.7)	0.362
**Smoking status** Non- or light smoker Current or former smoker	26 (9.7) 241 (90.3)	13 (8.6) 139 (91.4)	13 (11.3) 102 (88.7)	0.533
**ECOG PS** 0–1 2	227 (85) 40 (15)	136 (89.5) 16 (10.5)	91 (79.1) 24 (20.9)	0.024
**Histological subtype** Squamous Non-squamous	86 (32.2) 181(67.8)	49 (32.2) 103 (67.8)	37 (32.2) 78 (67.8)	1.000
**Treatment line** 1st line ≥2nd line	81 (30.3) 186 (69.7)	60 (39.5) 92 (60.5)	21 (18.3) 94 (81.7)	<0.001
**Immune-checkpoint blockade schedule** Monotheraphy Combination with chemotherapy Combination with anti-CTLA-4	209 (78.3) 33 (12.3) 25 (9.4)	112 (73.7) 24 (15.8) 16 (10.5)	97 (84.4) 9 (7.8) 9 (7.8)	0.082
**Treatment duration** median (range)	2.75 m (0.03–56.4, IQR 6.2)	4.8 m (0.03–56.4, IQR 9.8)	1.8 m (0.03–46.2, IQR 2.7)	<0.001
**Prednisone equivalent ≥10 mg/day use** No Yes	133 (49.8) 134 (50.2)	66 (43.4) 86 (56.6)	67 (58.3) 48 (41.7)	0.019
**Antibiotics use** No Yes	126 (47.2) 141 (52.8)	63 (41.4) 89 (58.6)	63 (54.8) 52 (45.2)	0.036

*irAEs, immune-related adverse events; IQR, interquartile range; ECOG PS, Eastern Cooperative Oncology Group performance status; CTLA-4, cytotoxic T-lymphocyte antigen 4*.

### Anti-PD-(L)1 Blockade Treatment and irAEs Characteristics

Eighty-one patients (30.3%) received anti-PD-(L)1 blockade agents as first line treatment, 131 (49.1%) as second line treatment and 55 (20.6%) as third line treatment or beyond. Anti-PD-(L)1 blockade agents were given alone (78.3%) or in combination with chemotherapy (12.3%) or with an anti-CTLA-4 agent (9.4%). Nivolumab (44.2%) and pembrolizumab (25.6%) were the most commonly used types of anti-PD-(L)1 blockade agents, followed by atezolizumab (17.2%).

One hundred and fifty-two patients (56.9%) experienced a total of 255 irAEs. The median number of irAEs per patient was one (range 0–5, IQR 1), and 64 patients (24%) experienced two or more irAEs. The most common irAEs was skin toxicity (35.6%), followed by diarrhea (16.5%) and hypothyroidism (10.2%). According to the CTCAE terminology, 149 irAEs (58.4%) were grade 1, 65 (25.5%) were grade 2, 33 (12.9%) were grade 3 and three (1.2%) were grade 4. There were five treatment-related deaths (2%): four due to pneumonitis and one due to hepatitis. IrAEs were more frequent in patients receiving immunotherapy in first line treatment (74.1%) than in second line treatment or beyond (49.5%) (*p* < 0.001). No differences were observed in the presence of grade ≥3 irAEs according to the treatment line (*p* = 0.342). A trend to a higher rate of grade ≥3 irAEs was also seen in patients receiving immune blockade combination regimens (50%) in contrast to single-agent anti-PD-1 and anti-PD-L1 (21.6 and 20%, respectively) (*p* = 0.058).

Endocrine toxicity was significantly higher with a combination of immune blockade agents (36%) than with single-agent anti-PD-1 (8.6%) or anti-PD-L1 (8.9%) (*p* = 0.034). A trend to a higher rate of pneumonitis was seen with combinations of immune blockade agents (20% vs. 6.5 and 10.7%) (*p* = 0.101), and a greater number of cases of arthritis was observed with anti-PD-1 blockade agents (11.3%) than with anti-PD-L1 (3.6%) or than with a combination of immune blockade agents (4%) (*p* = 0.099). Global median time to irAEs onset was 7.6 weeks (0.1–123.4, IQR 13.3). A description of irAEs and median onset time are detailed in Table 2.

**Table 2:** Description of immune-related adverse events.

**Types of irAEs**	**All patients**, ***n*** **=** **267 (%)**			**Median onset time (range), weeks**
	**All grades *n* = 255^**a**^ (95.5)**	**Grade 3–5 *n* = 41 (15.3)**	**irAEs requiring prednisone equivalent ≥10 mg/d^**d**^*n* = 63 (23.6)**	
**Cutaneous**Rash Pruritus	45 (17) 46 (17.2)	3 (1.1) 0	5 (2) 3 (1.1)	10.8 (0.3–145)
**Diarrhea**	42 (15.7)	6 (2.2)	8 (3)	8.9 (0.1–89.7)
**Endocrine dysfunction**Hypothyroidism Hyperthyroidism Adrenal insufficiency	26 (9.7)^e^ 6 (2.2) 4 (1.5)	1 (0.4) 0 2 (0.7)	1 (0.4) 2 (0.7) 3 (1.1)	16 (1.7–106)
**Pneumonitis**	23 (8.6)	12^b^ (4.5)	19 (7.1)	16.7 (0.9–189.4)
**Hepatitis**	14 (5.3)	4^c^ (1.5)	7 (2.6)	5.7 (0.4–33)
**Mucositis**	2 (0.7)	1 (0.4)	0	2.7 (1.6–3.9)
**Arthritis**	24 (9)	0	4 (1.5)	11 (0.3–123.4)
**Others**Hemolytic anemia Thrombocytopenia Flu-like Nephritis Vitiligo Pancreatitis Myopericarditis Myositis Vasculitis Aseptic meningitis Encephalitis Miasteniforme syndrome	23 (8.6) 1 2 4 4 1 1 2 3 1 1 2 1	12 (4.5) 1 2 0 1 0 1 2 1 0 1 2 1	11 (4.1) 1 1 0 2 0 1 1 1 1 1 2 0	Not calculated

*irAEs, immune-related adverse events*.

a *Total number of irAEs*.

b, c *Four cases of pneumonitis and one case of hepatitis were grade 5*.

d *High-dose steroid pulse therapy (metilprednisolone at 1g/day) for 3 days followed by metilprednisolone (1 to 2 mg/kg) treatment for several weeks was administered in one case of grade 3 colitis. No patient received other types of immunosupressors*.

e *Eleven patients required thyroid hormone replacement therapy*.

The median duration of treatment with anti-PD-(L)1 blockade agents was significantly longer in patients who experienced irAEs than in those who did not: 4.8 months (range 0.03–56.4, IQR 9.8) vs. 1.8 months (range 0.03–46.2, IQR 2.7) (*p* < 0.001). Comparisons between patients regarding the presence of irAEs can be found in Table 1.

Two hundred and eighteen patients (82%) discontinued treatment with anti-PD-(L)1 blockade agents. The most common reason given was progressive disease in 145 patients (66.5%), followed by the presence of irAEs in 44 patients (20.2%). Twenty-nine patients (13.3%) stopped treatment due to other causes, such as deterioration in their general condition or complications unrelated to disease progression. Pneumonitis (34.1%), endocrine dysfunction (29.5%), and diarrhea (22.7%) were the irAEs most frequently associated with treatment discontinuation. Thirty of the 44 patients who stopped anti-PD-(L)1 therapy due to toxicity presented irAEs grade ≥3 (68.2%). By the time of data analysis, 83.9% of irAEs had been resolved.

### Association Between irAEs and Treatment Outcomes

At the time of data analysis, the median follow-up time was 8.5 months (range 0.3–56.4, IQR 10.6) and the median duration of treatment with anti-PD-(L)1 blockade agents was 2.8 months (range 0.1–56.4, IQR 6.2). The median OS and PFS of the study population were 12.4 months (95% confidence interval [CI], 10.1–14.7) and 4.2 months (95% CI, 3.1–5.3), respectively. In first line setting, the median OS was 19.4 months (95% CI, 11.9–27.0) and the median PFS was 9.8 months (95% CI, 5.4–14.2). As expected, patients receiving anti-PD-(L)1 therapy as second line treatment or beyond had significantly poorer outcomes, with a median OS of 9.0 months (95% CI, 7.0–11.1) (HR 1.89; 95% CI, 1.29–2.79; *p* = 0.001) and a median PFS of 3.3 months (95% CI, 2.6–4.1) (HR 1.78; 95% CI, 1.27–2.51; *p* = 0.001). No differences were found between patients receiving anti-PD-(L)1 blockade agents in monotherapy or combination with anti-CTLA-4 or chemotherapy in first line setting, neither in terms of OS (*p* = 0.177) nor in PFS (*p* = 0.343).

**Figure 1:**
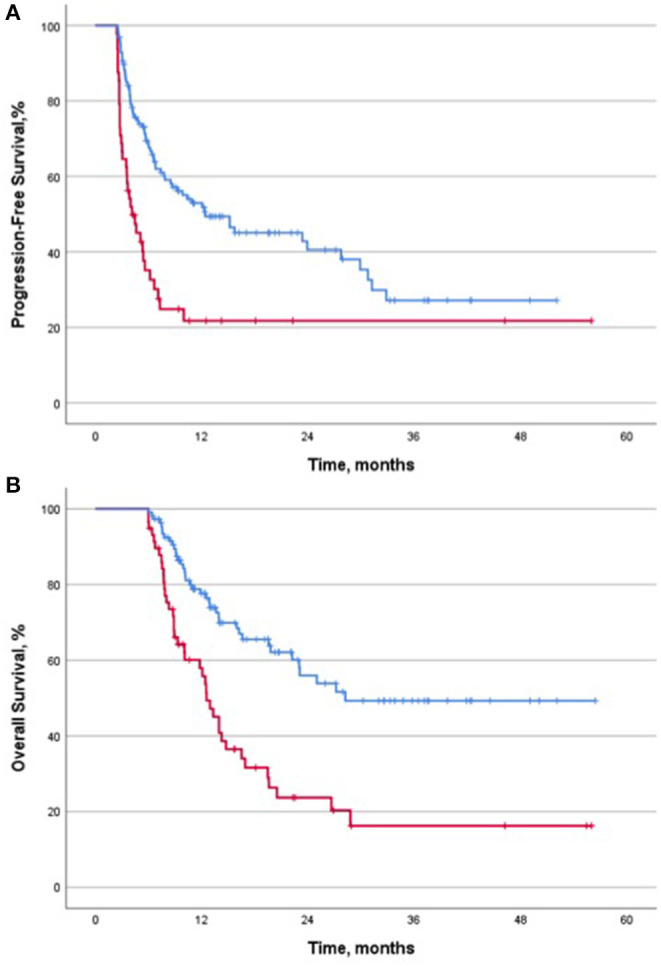
Landmark analysis according to the presence of irAEs. **(A)** Progression-free survival and the presence of irAEs (*n* = 175). (**B)** Overall survival and the presence of irAEs (*n* = 167). Kaplan-Meier curves with 2.4 months landmark analysis for progression-free survival **(A)** and 5.9 months for overall survival **(B)** in patients with or without irAEs. Abbreviations: PFS, progression-free survival; OS, overall survival; irAEs, immune-related adverse events; CI, confidence interval; HR, hazard ratio; m, months.

The landmark analysis showed that PFS was significantly longer in patients experiencing irAEs than in those without irAEs: 12.4 months (95% CI, 1.9–22.9) vs. 4.1 months (95% CI, 2.6–5.6), (HR 0.43; 95% CI, 0.28–0.64; *p* < 0.001). Similarly, OS among patients with irAEs was significantly higher: 28.2 months (95% CI, not achieved) vs. 12.5 months (95% CI, 10.8–14.2) (HR 0.38; 95% CI, 0.24–0.59; *p* < 0.001) (Figures 1A,B).

Six and 12 months landmark analyses were also performed to provide complementary information. The median OS at both time-points also favored patients with irAEs: 12.9 months (95% CI, 11.3–14.5) vs. 28.2 months (95% CI not calculated) (HR 0.39; 95% CI, 0.25–0.61; *p* < 0.001), and 19.6 months (95% CI, 15.2–23.9) vs. not reached (HR 0.33; 95% CI, 0.17–0.64; *p* = 0.001), respectively. Landmark analyses for PFS at 6 and 12 months could not be calculated since no event happened after these time-points in the no-irAEs group.

Of note, the ORR was significantly higher in patients who experienced irAEs than in those without irAEs: 48.6 vs. 22.8% (odds ratio [OR] 0.31; 95% CI, 0.18–0.55; *p* < 0.001). DCR was also significantly better when irAEs were present: 77.1 vs. 39.6% (OR 0.20; 95% CI, 0.11–0.34; *p* < 0.001).

The landmark analysis was also applied when comparing ORR regarding the development of irAEs. Landmark analysis at 8 and 10 weeks showed that ORR was significantly higher in the irAEs group of patients. At 8 weeks, 54.8 vs. 28% (OR 0.32; 95% CI, 0.15–0.68; *p* = 0.004) and at 10 weeks, 62.3 vs. 36.7% (OR 0.35; 95% CI, 0.14–0.85; *p* = 0.028). However, no differences were detected when greater time-point were used, probably because the progressive decline in number of patients who show first response later in time (Table S1).

Though median time to response was slightly shorter in the no-irAEs group [8 weeks (1.3–122.6, IQR 5.3) vs. 9.8 weeks (1.9–117.4, IQR 11.3) (*p* = 0.004)], DoR was significantly longer in patients with irAEs: 6.1 months (range 0.5–50, IQR 10.6) vs. 2.6 months (range 0.2–51.9, IQR 3.8) (*p* < 0.001). As mentioned previously, 44 patients (22.2%) discontinued treatment due to irAEs. Within this group, 29 patients (65.9%) did not progress after stopping immunotherapy, in contrast to the 28.7% (64/223) of patients in the group of patients who did not discontinue treatment due to toxicity (*p* < 0.001).

### Association Between the Use of Corticosteroids and Efficacy

The most commonly used types of corticosteroids were prednisone (39.7%) and dexamethasone (34.9%). The median dose of prednisone equivalent was 50 mg daily (range 5–1,250 mg, IQR 53.4). The median duration of corticosteroid treatment was 59 days (range 0.5–83.0, IQR 159). No differences in corticosteroid usage were observed in patients receiving first line therapy (53.1%) vs. second line or beyond (55.4%) (*p* = 0.790).

One hundred and forty-six patients (54.7%) received corticosteroids during therapy with anti-PD-(L)1 blockade agents, of whom 134 patients (91.8%) required ≥10 mg of prednisone equivalent per day: 59 patients (44%) for the treatment of irAEs, and 75 patients (56%) for the management of cancer-related symptoms, including asthenia (8.2%) and anorexia (6.3%), symptomatic bone metastases (13.4%), symptomatic brain metastases (36.3%), dyspnea (14.8%), and chronic obstructive pulmonary disease (COPD) management (21%). No other chronic illness required steroid therapy in our study population. Only seven patients started corticosteroids within the 30 days before immunotherapy initiation, and all patients continued corticosteroids therapy during anti-PD-(L)1 therapy (Figure S1). Patients receiving corticosteroids for cancer-related symptoms presented significant differences compared to the rest of the population: there was a higher proportion of patients with ECOG PS 2 (24.1 vs. 10.9%; *p* = 0.009) receiving second line therapy or beyond (83.1 vs. 63.6%; *p* = 0.001) or as a single-agent instead of a combination regimen (90.4 vs. 72.8%; *p* 0.002). No differences were observed regarding the presence of liver or brain metastases. Interestingly, patients receiving corticosteroids for cancer-related symptoms presented a lower incidence of irAEs (38.6 vs. 65.2%; *p* < 0.001) (Table 3).

**Table 3:** Comparison of patient characteristics between patients receiving corticosteroids for cancer-related symptoms and patients not receiving corticosteroids or received corticosteroids for management of irAEs^*^.

**Category**	**Total *n* = 267 (%)**	**Corticosteroids for cancer-related symptoms *n* = 83 (%)**	Corticosteroids for irAEs or prednisone equivalent <10 mg/d *n = 184(%)	***P*-value**
**Gender**Male Female	186 (69.7) 81 (30.3)	60 (72.3) 23 (27.7)	126 (68.5) 58 (31.5)	0.568
**Age** median (range)	66.1 years (26.7–85.2, IQR 14.3)	63.6 years (38.8–85.2, IQR 13.3)	66.7 years (26.7–83.7, IQR 14.2)	0.742
**Smoking status**Non- or light smoker Current or former smoker	26 (9.7) 241 (90.3)	9 (10.8) 74 (89.2)	17 (9.2) 167 (90.8)	0.662
**ECOG PS**0–1 2	227 (85) 40 (15)	63 (75.9) 20 (24.1)	164 (89.1) 20 (10.9)	0.009
**Histological subtype**Squamous Non-squamous	86 (32.2) 181(67.8)	25 (30.1) 58 (69.9)	61 (33.2) 123 (66.8)	0.673
**Treatment line**1st line ≥2nd line	81 (30.3) 186 (69.7)	14 (16.9) 69 (83.1)	67 (36.4) 117 (63.6)	0.001
**Immune-checkpoint blockade schedule**Monotherapy Combination with chemotherapy Combination with anti-CTLA-4	209 (78.3) 33 (12.3) 25 (9.4)	75 (90.4) 3 (3.6) 5 (6)	134 (72.8) 30 (16.3) 20 (10.9)	0.002
**Presence of irAEs**No Yes	115 (43.1) 152 (56.9)	51 (61.4) 32 (38.6)	64 (34.8) 120 (65.2)	<0.001
**Presence of brain metastases**No Yes	225 (84.3) 42 (15.7)	65 (78.3) 18 (21.7)	160 (87) 24 (13)	0.101
**Presence of liver metastases**No Yes	226 (84.6) 41 (15.4)	67 (80.7) 16 (19.3)	159 (86.4) 25 (13.6)	0.272
**Prednisone equivalent ≥10 mg/day use**No Yes	133 (49.8) 134 (50.2)	8 (9.6) 75 (90.4)	125 (67.9) 59 (32.1)	<0.001
**Antibotic use**No Yes	126 (47.2) 141 (52.8)	32 (38.6) 51 (61.4)	94 (51.1) 90 (48.9)	0.064

*irAEs, immune-related adverse events; IQR, interquartile range; ECOG PS, Eastern Cooperative Oncology Group performance status; CTLA-4, cytotoxic T-lymphocyte antigen 4*.

* *Includes patients that did not received corticosteroids*.

**Figure 2:**
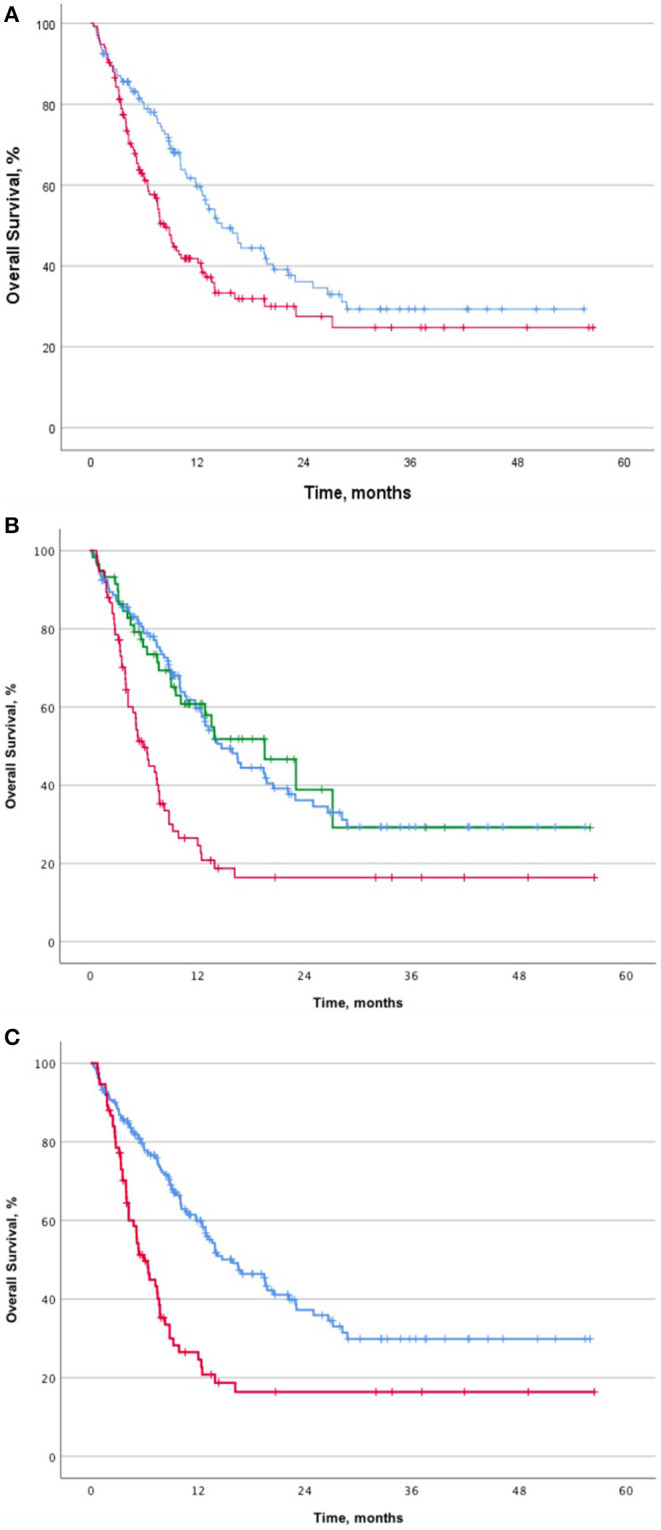
Survival analysis with regard to corticosteroid use (≥10 mg of prednisone equivalent daily). **(A)** Overall survival and the use of ≥10 mg of prednisone equivalent daily (*n* = 267). **(B)** Overall survival and the reason for corticosteroid use (*n* = 267). **(C)** Overall survival and corticosteroid use (*n* = 267). Abbreviations: PFS, progression-free survival; OS, overall survival; irAEs, immune-related adverse events; PDNe, prednisone equivalent; CI, confidence interval; HR, hazard ratio; m, months. Kaplan-Meier curves of patients treated with anti-PD-(L)1 blockade agents on the basis of reported corticosteroid usage (≥10 mg of prednisone equivalent) in terms of OS **(A)**. OS comparison in patients who did not received any corticosteroids, those who received them for irAEs treatment or cancer-related symptoms management **(B)**. OS comparison according to the use of corticosteroids: for management of cancer-related symptoms and the rest of the population **(C)**. ^a^Use of ≥10 mg of prednisone equivalent daily for irAEs management. ^b^Use of ≥10 mg of prednisone equivalent daily for cancer-related symptoms.

Median OS was significantly longer in the group of patients that received <10 mg prednisone equivalent daily or no corticosteroids (*n* = 133) than in the group of patients that received ≥10 mg prednisone equivalent daily (*n* = 134): 14.7 months (95% CI, 11.1–18.3) vs. 8.3 months (95% CI, 6.9–9.8) (HR 0.66; 95% CI, 0.48–0.90; *p* = 0.010) (Figure 2A). No differences in PFS were observed. Median OS was significantly shorter in patients receiving ≥10 mg prednisone equivalent daily for cancer-related symptoms (*n* = 75) than in the rest of the study population (patients who did not receive corticosteroids or <10 mg prednisone equivalent daily and those who received them for the management of irAEs, *n* = 192): 6 months (95% CI, 4.4–7.5) vs. 15.9 months (95% CI, 11.2–20.7) (HR 2.28; 95% CI, 1.63–3.20; *p* < 0.001). No differences in terms of PFS were observed.

No differences in OS were found between patients who started corticosteroids for cancer-related symptoms in the 30 days before starting immunotherapy or after starting anti-PD-(L)1 blockade: 5.2 months (95% CI, 0.3–4.6) vs. 6.4 months (95% CI, 1.1–4.3) (*p* = 0.898).

It is important to highlight that no significant differences were observed in OS between patients who received no corticosteroids or <10 mg prednisone equivalent daily and those who received ≥10 mg for the management of irAEs (*p* = 0.314) (Figures 2B,C). However, when analyzing the outcomes of patients with irAEs, median OS was greater in patients receiving <10 mg of prednisone equivalent daily than those receiving ≥10 mg for toxicity management: not reached vs. 19.5 months (95% CI, 10.7–28.4) (HR 2.05; 95% CI, 1.13–3.73; *p* = 0.016).

In our study, a duration of corticosteroids ≥30 days correlated with a better outcome in terms of OS: 12.1 months (95% CI, 8.3–15.8) vs. 4.6 months (95% CI, 2.4–6.7) (*p* = 0.001). The same happened when the cut-off point was changed to 15 days: 9.3 months (95% CI, 5.3–13.2) vs. 5.1 (95% CI, 2.4–7.9) (*p* = 0.007). No differences in terms of PFS were detected (*p* = 0.746 and *p* = 0.726 for a cut-off of 30 and 15 days, respectively). However, when analyzing results in terms of corticosteroid use, both OS and PFS were higher in patients receiving ≥10 mg prednisone equivalent for irAEs management. In terms of OS, patients treated with ≥10 mg of prednisone equivalent for irAEs management during ≥30 days presented the highest survival rates: 23 months (95% CI, 11.5–34.6) (*p* 0.001). Notably, no differences were detected when steroid therapy was given for cancer-related symptoms.

### Association Between the Use of Antibiotics and Efficacy

One hundred and forty-one patients (52.8%) received antibiotics. Quinolone (37.6%) and penicillin (33.3%) were the most commonly used groups of antibiotics. Of note, the group of patients experiencing irAEs received significantly more antibiotics (58.6 vs. 45.2%, *p* = 0.036).

However, no relation was found between the use of antibiotics and efficacy of anti-PD-(L)1 blockade agents, with a median OS of 10.2 months (95% CI, 6.4–13.9) in patients receiving antibiotics vs. 12.5 months (95% CI, 9.9–15.0) in patients not receiving antibiotics (*p* = 0.924), and a median PFS of 3.8 months (95% CI, 0.9–1.9) vs. 4.4 months (95% CI, 0.7–2.9), respectively (*p* = 0.454).

### Multivariable Analysis

Multivariable analyses revealed that the presence of irAEs was the variable most strongly associated with a better response rate and OS (Table 4A and Table S2A), with an OR and HR of 0.36 and 0.32, respectively. In addition, in the multivariable analysis of types of irAEs, cutaneous, endocrinological, and rheumatological irAEs were found to be significantly associated with increased ORR and OS (Table 4B and Table S2B). Pruritus and arthritis were the irAEs subtypes with the lowest HR.

**Table 4:** Multivariable analysis of overall response rate according to clinical features (A) and type of irAEs (B).

**Variable**	**Odds ratio**	**95% CI**	***P-*value**
**(A). Overall response rate and clinical features (*****n*** **=** **267)**			
**Presence of irAEs**No Yes	0.36	0.20–0.65	0.001
**Treatment line**1st line ≥2nd line	2.41	1.36–4.28	0.003
**(B). Overall response rate and type of irAEs (*****n*** **=** **267)**			
**Rash**No Yes	0.34	0.17–0.69	0.002
**Endocrine dysfunction**No Yes	0.38	0.18–0.81	0.012
**Arthritis**No Yes	0.28	0.11–0.72	0.008

*CI, confidence interval; irAEs, immune-related adverse events*.

Variables such as ECOG PS ≥ 2, the presence of liver metastasis, the use of corticosteroids, and receiving anti-PD-(L)1 therapy as a second line treatment or beyond were related to poorer outcomes, specially regarding to OS (Table 2A). The HR for OS regarding corticosteroid use for cancer-related symptoms was >2 (HR = 2.40), though it gave a result of 1.81 when corticosteroids were used for irAEs management. Notably, no association was found between OS or ORR and the presence of brain metastasis. No differences were found regarding the grade of irAEs (grade 1–2 vs. grade 3–4 irAEs, excluding grade 0), nor in terms of OS (*p* = 0.198) nor in regard to ORR (*p* = 0.349).

## Discussion

As far as we are aware, this is one of the largest studies to assess the association between the presence of irAEs and the efficacy of anti-PD-(L)1 blockade agents in advanced NSCLC patients. Statistically significant differences were observed in terms of OS, PFS, ORR and DoR between patients experiencing irAEs and those who did not. Of note, a landmark analysis was performed to minimize the immortal time bias potentially associated with time-dependent factors such as the development of irAEs.

In our study the incidence of irAEs was 56.9%, which is higher than previously reported (13–23). This could be explained mainly because this is a real-world data study and, in addition, 30.3% of patients received anti-PD-(L)1 blockade agents in a first line setting. Patients receiving anti-PD-(L)1 blockade agents as a second line therapy or beyond experienced significantly fewer irAEs than those treated in the first line. These findings are in line with those observed in KEYNOTE-010 and KEYNOTE-024 trials showing a higher rate of toxicity in treatment naïve patients who are probably less immunosuppressed than pre-treated patients (5, 8). A significantly higher rate of endocrinological toxicity was observed with the combination of immune blockade agents. This was also reported by a group of experts in endocrinopathies. In their review, hypophysitis was more common with anti-CTLA-4 agents, whereas thyroid dysfunction was more frequent with anti-PD-1 agents. The combination of these agents appeared to increase the risk of immune-related endocrinopathies, which may be related to a more frequent association between CTLA-4 polymorphisms and autoimmune endocrinopathies in comparison with PD-1 polymorphisms (31).

The landmark analysis showed longer PFS and OS, and more importantly, a greater ORR, in patients experiencing irAEs, which corroborates the influence exerted by the development of immune-mediated toxicity on immunotherapy efficacy. The longer duration of treatment in patients experiencing irAEs might be explained by a lower percentage of treatment discontinuation in this group (75 vs. 91%, *p* = 0.001). This raises the question of whether the survival advantage attributed to the presence of irAEs is a reflection of the increased toxicity associated with a longer duration of treatment or a direct result of the irAEs themselves. Recently, both prospective and retrospective data have suggested that this relation is independent of guarantee-time bias, mainly because the majority of patients developed irAEs within the first 2–8 weeks after treatment initiation, supporting the predictive value of irAEs over treatment duration (22, 23).

The presence of irAEs itself was the strongest variable associated with better outcomes in a multivariable analysis, both according to ORR (OR 0.36; 95% CI, 0.20–0.65; *p* = 0.001) and OS (HR 0.32; 95% CI, 0.22–0.46; *p* < 0.001). Additionally, endocrine dysfunction, rash/pruritus, and arthritis were significantly associated with increased ORR and OS. Pruritus and arthritis presented the most favorable HR. Consistent with our findings, two studies have also suggested that thyroiditis and skin toxicity are related to longer OS (32, 33). Of note, no association was found between OS or ORR and the severity of irAEs.

Corticosteroids are the mainstay in the management of irAEs, though they are also a common symptomatic treatment in advanced NSCLC patients. Prednisone equivalent ≥10 mg daily for the symptomatic treatment of cancer-related symptoms at the time of initiation or during anti-PD-(L)1 blockade treatment was associated with significantly poorer outcomes than for patients who did not receive corticosteroids or those who received them to manage irAEs. These results are in line with those reported recently in a study carried out on 640 patients with advanced NSCLC receiving single-agent anti-PD-(L)1 blockade treatment (25). In that publication, a multivariable analysis including smoking status, ECOG, and history of brain metastases showed that baseline corticosteroid use was significantly associated with decreased ORR, PFS, and OS. Our multivariable analysis assessed similar prognostic factors, but also included the treatment line as a variable that could influence outcomes. Corticosteroid use for palliation of cancer-related symptoms and anti-PD-(L)1 therapy in the second line or beyond were strong, independent variables associated with a poorer outcome. Patients receiving corticosteroids for cancer-related symptoms showed a higher percentage of patients with an ECOG PS of 2 and treatment in the second line or beyond with single-agent. On the whole, these findings suggest that baseline corticosteroid use may simply identify a group of patients with a higher volume or more aggressive disease or with basal illnesses that worsen cancer prognosis. The same inference has been made in a recently published study in which the authors concluded that the worst outcome associated with ≥10 mg of prednisone equivalent daily at the time of immunotherapy seemed to be driven by a poor-prognosis subgroup of patients who received corticosteroids as a palliative treatment (26). Moreover, previous data suggest that the positive correlation between the presence of irAEs and efficacy of anti-PD-(L)1 blockade agents in advanced NSCLC is not hampered by the use of corticosteroids for the treatment of irAEs, concluding that its use in this context should not be restricted for fear of loss of any outcome advantage. In contrast, our study showed a greater median OS in patients receiving <10 mg of prednisone equivalent than those receiving ≥10 mg for toxicity management. The median duration of ≥10 mg prednisone equivalent had a significant positive impact on the efficacy of anti-PD-(L)1 blockade agents when given for irAEs management, but this finding might be explained by irAEs itself playing a role as a confounding factor.

Regarding the implications of the use of antibiotics, no relation was found between the use of antibiotics (3 months before, during or 3 months after the end of anti-PD-(L)1 therapy) and immunotherapy efficacy. These results differ from those observed in a cohort of 249 advanced NSCLC, renal cell and urothelial carcinoma patients, in which 28% of patients received antibiotics within 2 months before or 1 month after the initiation of anti-PD-(L)1 blockade agents, for whom both PFS and OS were significantly shorter (34). However, those results were based on 69 out of 249 patients, so although informative, they are insufficient to draw any firm conclusion. In addition, the different time period over which the use of antibiotics was analyzed makes it difficult to compare results. The prospective analysis of the effect of antibiotics on the efficacy of anti-PD-(L)1 therapy might help to understand the relation between them and the period of time when the use of antibiotics might be discouraged.

Anti-PD-(L)1 therapy interruption due to irAEs is an issue that concerns us all. In our study, the 65.9% of patients who discontinued treatment due to irAEs and did not progress contrasts with the 28.7% of patients who did not interrupt immunotherapy due to toxicity and did not progress. These results are in line with a *post-hoc* analysis of the Checkmate-067 trial in patients with advanced melanoma, in which both PFS and OS were similar after 4 years regardless of discontinuation of treatment due to irAEs (35). Taken together, these data suggest that treatment interruption due to irAEs does not seem to compromise the efficacy of anti-PD-(L)1 blockade agents.

This study has several limitations that could be addressed in future research. First, its retrospective design and the need for a longer follow-up period to fully assess long-term outcomes. Second, the heterogeneity of treatment strategies included in this study, which may influence the efficacy of immunotherapy and the frequency of irAEs. Third, the low frequency of some irAEs subtypes may limit the evaluation of their relationship with efficacy. A prospective study including a larger cohort of patients with the same treatment strategy would help to overcome those limitations and adequately assess the real impact of corticosteroids use by accounting for potential confounding factors.

In conclusion, our results indicate that the presence of irAEs is associated with anti-PD-(L)1 blockade agents efficacy in patients with advanced NSCLC. This is one of the most extensive studies to date to reveal an association between the presence of irAEs and the efficacy of immunotherapy in advanced NSCLC when landmark and multivariable analyses are applied. Corticosteroid use of ≥10 mg of prednisone equivalent daily was associated with significantly poorer outcomes when given for patients' cancer-related symptoms. No significant differences were observed in terms of efficacy between patients that did not receive corticosteroids or who received <10 mg prednisone equivalent daily and those who received ≥10 mg for the management of irAEs. No relation was found between antibiotics and outcomes of anti-PD-(L)1 blockade agents.

## Data Availability Statement

The datasets generated for this study are available on request to the corresponding author.

## Ethics Statement

The studies involving human participants were reviewed and approved by Hospital de la Santa Creu i Sant Pau. The patients/participants provided their written informed consent to participate in this study.

## Author Contributions

MR and MM contributed to the conception and design of the study, data acquisition, statistical analysis, interpretation of the data, and writing of the manuscript. JM and RG-C contributed to the acquisition and interpretation of the data and revision of the manuscript. IG and LC contributed to the statistical analysis and interpretation of the data. JS, GA, PG, IS, ABarb, and ABarn contributed to the acquisition of the data. All authors reviewed and approved the final version of the manuscript. All authors contributed to the article and approved the submitted version.

## Conflict of Interest

RG-C reports personal fees and other from Roche, BMS, MSD, Pfizer, Lilly, AstraZeneca, Takeda, Novartis, Boeringher Ingelheim, during the conduct of the study. ABarb reports non-financial support from MSD, Roche, AstraZeneca, BMS, outside the submitted work. ABarn reports grants from Roche, Novartis, Pfizer, BMS, AstraZeneca, Lilly, during the conduct of the study, personal fees from Novartis, Roche, Pfizer, Genomic Health, Pierre Fabre, AstraZeneca, EISAI, outside the submitted work. MM reports personal fees and other from Viforpharma, Roche, BMS, MSD, Pfizer, Lilly, AstraZeneca, Takeda, Novartis, Boeringher Ingelheim, during the conduct of the study. The remaining authors declare that the research was conducted in the absence of any commercial or financial relationships that could be construed as a potential conflict of interest. The handling editor declared a past co-authorship with several of the authors IS and MM.
