# Supplementary material for: Immune-Related Adverse Events and Corticosteroid Use for Cancer-Related Symptoms Are Associated With Efficacy in Patients With Non-small Cell Lung Cancer Receiving Anti-PD-(L)1 Blockade Agents
Source: Front Oncol. 2020 Sep 7;10:1677. doi: 10.3389/fonc.2020.01677 (PMC7505083; doi:10.3389/fonc.2020.01677)
Supplement: Supplementary file 1 [file Data_Sheet_1.docx]

**Supplementary material**

**Figure S1. Flow chart detailing corticosteroid usage: reasons and doses.**

Abbreviations: irAEs, immune-related adverse events.

To analyze efficacy according to the dose of corticosteroids patient were divided into two groups: those receiving prednisone equivalent ≥10mg daily and those receiving prednisone equivalent ≤ 10 mg daily, which includes patients who did not receive corticosteroids.

**Table S1. Landmark analysis of ORR according to the presence of irAEs**

| **Landmark analyses** | **ORR** | | ***Odds ratio***  **(95% CI)** | ***P* value** |
| --- | --- | --- | --- | --- |
|  | ***irAEs group*** | ***no-irAEs group*** |  |  |
| 8 weeks  (n = 134) | 54.8% | 28% | 0.32  (0.15-0.68) | 0.004 |
| 10 weeks  (n = 99) | 62.3% | 36.7% | 0.35  (0.14-0.85) | 0.028 |
| 12 weeks  (n = 69) | 61.4% | 50% | 0.63  (0.18-2.19) | 0.527 |
| 16 weeks  (n = 44) | 100% | 72.5% | 1.37  (1.14-1.67) | 0.558 |

Abbreviations: ORR, overall response rate; irAEs, immune-related adverse events; CI, confidence interval.

**Table S2. Multivariable analysis of overall survival according to clinical features (A) and type of irAEs (B)**

**A. Overall survival and clinical features (n = 267)**

| **Variable** | ***Hazard ratio*** | **95% CI** | ***P* value** |
| --- | --- | --- | --- |
| **ECOG PS**  0-1  ≥2 | 1.67 | 1.09-2.57 | 0.019 |
| **Presence of liver metastases**  No  Yes | 1.84 | 1.20-2.80 | 0.005 |
| **Presence of irAEs**  No  Yes | 0.32 | 0.22-0.46 | <0.001 |
| **Corticosteroids use**  (Prednisone equivalent ≥10mg/day)  No  For irAEs management  For cancer-related symptoms | 1.28  2.40 | 0.66-2.47  1.37-4.22 | 0.466  0.002 |
| **Treatment line**  1st line  ≥2nd line | 1.56 | 1.04-2.34 | 0.033 |

**B. Overall survival and type of irAEs (n = 267)**

| **Variable** | ***Hazard ratio*** | **95% CI** | ***P* value** |
| --- | --- | --- | --- |
| **Rash**  No  Yes | 0.42 | 0.23-0.75 | 0.003 |
| **Pruritus**  No  Yes | 0.26 | 0.14-0.49 | <0.001 |
| **Endocrine dysfunction**  No  Yes | 0.59 | 0.34-1.00 | 0.050 |
| **Arthritis**  No  Yes | 0.26 | 0.10-0.62 | 0.003 |

Abbreviations: CI, confidence interval; ECOG PS, Eastern Cooperative Oncology Group performance status; irAEs, immune-related adverse events.
